# Monitoring of haematopoietic stem cell mobilization by targeted DNA methylation analysis

**DOI:** 10.1111/bjh.70446

**Published:** 2026-03-17

**Authors:** Wouter H. G. Hubens, Anke Diehlmann, Patrick Wuchter, Wolfgang Wagner

**Affiliations:** ^1^ Institute of Stem Cell Biology University Hospital of RWTH Aachen Aachen Germany; ^2^ Helmholtz Institute for Biomedical Engineering RWTH Aachen University Aachen Germany; ^3^ Institute of Transfusion Medicine and Immunology, Medical Faculty Mannheim Heidelberg University Heidelberg Germany; ^4^ German Red Cross Blood Service Baden‐Württemberg—Hessen Mannheim Germany; ^5^ Center for Integrated Oncology Aachen Bonn Cologne Düsseldorf (CIO ABCD) Aachen Germany

**Keywords:** DNA methylation, epigenetic quantification, haematopoietic stem cells, mobilization

To the Editor,

Quantification of CD34+ haematopoietic stem and progenitor cells (HSPCs) is essential for evaluating the efficiency of mobilization, particularly for stem cell apheresis. In fact, HSPC counts in the transplant are a good indicator for consecutive engraftment after autologous or allogeneic haematopoietic stem cell transplantation.^1^ The standard CD34+ cell dose is generally considered to be 2 to 5 × 10^6^ cells/kg and is determined by flow cytometric analysis.^1^, ^2^ For determining the peak of CD34+ cell mobilization during the mobilization phase, fresh samples of peripheral blood are required. Thus, patients need to travel to the clinic for several days to determine the optimal time point for leukapheresis, whereas for allogeneic donors usually a fixed scheme of 10 μg granulocyte‐colony stimulating factor (G‐CSF)/kg body weight (bw) sc/day is applied for 4 days with leukapheresis starting at the morning of day 5. Here, we describe an alternative approach based on deoxyribonucleic acid (DNA) methylation.

During haematopoietic differentiation, the DNA methylation patterns are modified in a cell type‐specific manner, which can be used to deconvolute the leucocyte composition in blood.^3^ DNA methylation is a reversible epigenetic modification that, in humans, mainly occurs at cytosine nucleotides that is followed by a guanine (CpG sites). While many epigenetic estimates of the leucocyte composition are usually based on hundreds of CpGs,^4^ we previously demonstrated that this method can be further refined to a targeted analysis of individual cell type‐specific CpG sites, which accurately reflected the proportion of monocytes, granulocytes and lymphocyte subsets (CD4 T cells, CD8 T cells, B cells and natural killer cells).^5^, ^6^, ^7^ In contrast to conventional methods, this epigenetic quantification enables ‘remote’ analysis of dried blood spots obtained by a finger prick.^8^

In continuation of this work, we recently sought to identify DNA methylation biomarkers to quantify CD34+ HSPCs using publicly available Illumina Bead Chip Microarray data (Infinium Human Methylation 450 K BeadChip and Infinium MethylationEpic v1.0; Tables S1 and S2), as described in detail before.^7^, ^9^ In brief, the best suited candidate CpGs to discern CD34+ cells from other leucocyte subsets were identified based on low variance in DNA methylation between samples and had a high difference in DNA methylation between HSPCs versus all other cell types. Based on those criteria, we picked five promising targets within the genes SP140 nuclear body protein (*SP140*; cg17607231), CD48 molecule (*CD48*; cg13311440), nucleated factor of activated T cells 1 (*NFATC1*; cg11977716), myosin 1D (*MYO1D*; cg00164282) and serine/threonine kinase 17a (*STK17A*; cg17707057).

In the current study, we aimed to validate these epigenetic biomarkers and develop an epigenetic HSPC predictor, using peripheral blood samples obtained from 56 healthy donors of the Transfusion Medicine department at the Uniklinik RWTH Aachen and cryopreserved leukapheresis material from 172 allogeneic stem cell donors, that have been mobilized with G‐CSF (10 μg/kg bw sc/day for 4 consecutive days prior to leukapheresis) at the Institute of Transfusion Medicine and Immunology in Mannheim. The samples were collected in accordance with the Declaration of Helsinki, including an informed consent from all donors, and analysed anonymously. Samples were processed for targeted DNA methylation analysis as described in Supporting Information (Figure S1A). In short, genomic DNA was isolated (QIAamp DNA mini kit, Qiagen) and treated with bisulphite to convert unmethylated cytosines into uracil (EZ DNA Methylation Kit; Zymo research). Using fluorescently labelled probes designed to specifically bind to either methylated or unmethylated DNA (Table S3), we could infer methylation levels by digital polymerase chain reaction (QIAcuity One, Qiagen; Figure S1B).

Initially, we tested all five candidate CpGs in a small cohort of donors (*n* = 8). Except for the CpG in *NFATC1*, DNA methylation levels clearly correlated with CD34+ cell counts obtained via flow cytometry (Figure S1C). Furthermore, the DNA methylation values correlated between the individual CpGs, despite being on completely different chromosomes (Figure S1D), particularly for the CpGs in *SP140* and *MYO1D* (Pearson's correlation *r* = 0.998; Figure S1E). Although this sample size is not sufficient to exclude the suitability of any of the five CpGs as a potential biomarker, we decided to reduce the amount of CpGs to a maximum of three, as this could even be multiplexed in a single well, using the six optical channels installed in the digital PCR machine (each CpG needs two channels to infer methylated and unmethylated strands). Based on the slightly lower correlation for *NFATC1* and the strong inter‐CpG correlation for *MYO1D*, we therefore arbitrarily excluded these CpGs for further analysis.

The DNA methylation at the remaining three CpGs in the genes *SP140*, *CD48* and *STK17A* was subsequently measured in 56 non‐mobilized and 172 mobilized blood samples, and the levels were significantly higher in the mobilized samples (Figure 1A). To estimate CD34+ HSPC fractions based on these methylation values, we randomly selected 50 G‐CSF‐treated donors to train a multiple linear regression model (Figure 1B; Table S4), which was tested on the remaining samples. For the individual CpGs, the correlation of DNA methylation with CD34+ counts was rather moderate, which can be attributed to the very low cell fractions. However, the correlation significantly improved when we combined the three CpGs into a multilinear regression model (*r* = 0.57). Thus, the combination of several CpGs can outbalance some of the technical noise, as previously observed for epigenetic age predictions.^10^

**FIGURE 1:**
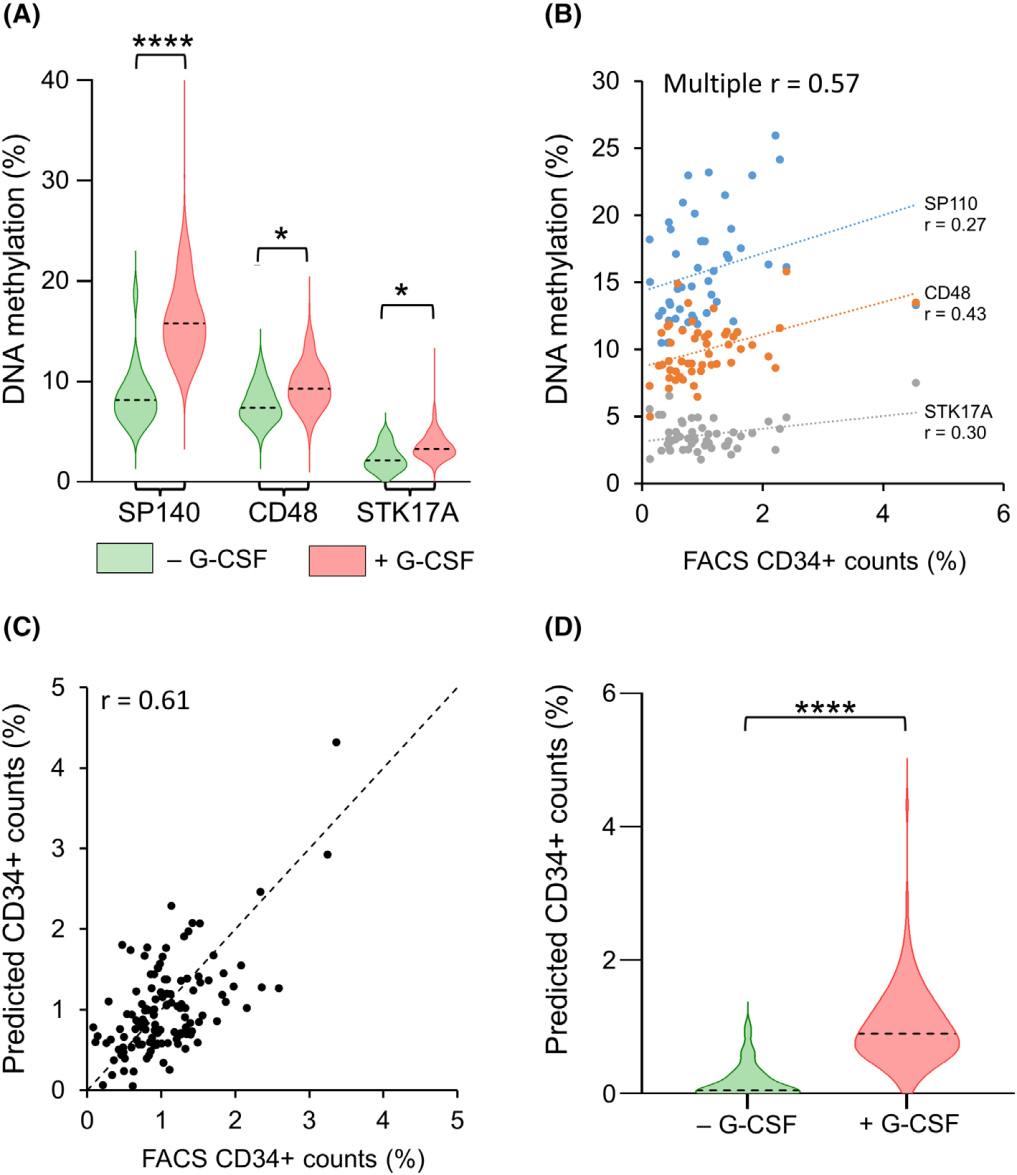
Epigenetic quantification of CD34+ cells in blood. (A) DNA methylation levels of the three candidate CpGs in peripheral blood of donors untreated (*n* = 56) or treated with granulocyte‐colony stimulating factor (G‐CSF) (*n* = 172). (B) Fifty randomly selected G‐CSF‐treated donors were withheld to train a multilinear regression model. (C) Correlation between predicted CD34+ counts and measurements obtained with the BD FACSLyric cytometer in the independent validation set. (D) Epigenetically predicted HSPC counts between untreated and treated donors. ****p < 0.001 and *p < 0.05 in Kruskal–Wallis with False Discovery Rate correction (A) or Mann–Whitney *U*‐test (D). Pearson correlation *r* is provided (B, C).

Despite CD34+ cells being only a very small fraction of the leucocytes, the predicted values and flow cytometric measurements showed a significant correlation (Figure 1C; *r* = 0.61, *p* < 0.001). Furthermore, our model could predict that the HSPC fraction was significantly higher in G‐CSF‐treated donors, in line with the CD34+ mobilization (Figure 1D). To assess if our assay might be clinically informative for successful mobilization, we performed a receiver operating characteristics analysis, which suggests that HSPC counts higher than 0.4% are indicative of successful G‐CSF treatment with a 93% specificity and 86% sensitivity (Figure S2; area under the curve in a receiver operating characteristic analysis of 0.94). Further validation is required to assess if this would be a suitable strategy to determine the optimal apheresis time point. It is also conceivable that, with other (or additional) CpGs, the model can be further improved for these specific clinical applications.

A limitation of our method is that we quantified relative CD34+ fractions, whereas absolute numbers of CD34+ cells per kg body weight are used as a readout for successful mobilization. Alternatively, to our proposed relative counts, it is also possible to estimate the absolute number of leucocytes based on the DNA content—either as direct measurement of DNA concentration or by relative amount as compared to a reference DNA.^6^ However, this approach was not applicable for our samples because they were cryopreserved, and upon thawing, there are notoriously changes in the amount of leucocytes.^11^ In future experiments, using a fixed amount of fresh blood (e.g. 3x10^‐5^ L), which might be sampled by a finger prick and dried on filter paper, can circumvent this issue.^7^

Taken together, our study provides the first promising proof of concept that targeted DNA methylation analysis can quantify CD34+ cells in peripheral blood upon mobilization by G‐CSF. Although our method is unlikely to replace the routine monitoring of mobilization with the gold standard of flow cytometry, it may ease determining the day for apheresis and thereby reduce the number of visits for the donor. As our approach can be performed with dried blood, a donor could for instance collect samples via a finger prick for several days on a daily basis and send this for epigenetic analysis. The procedure of the measurement could be performed within 6 h. As soon as the epigenetic HSPC counts are clearly on the rise, the donor is called for a single visit to validate these counts by flow cytometry and subsequent apheresis.

## AUTHOR CONTRIBUTIONS

Wouter H. G. Hubens was involved in the conceptualization of research, carried out the measurements, performed the data analysis and wrote the initial draft. Anke Diehlman provided essential leukapheresis material, performed flow cytometry cell counts and provided critical feedback for the manuscript. Patrick Wuchter provided funding for the essential leukapheresis material, performed flow cytometry cell counts and provided critical feedback for the manuscript. Wolfgang Wagner was involved in the conceptualization of research, organizing funding and critically revising the manuscript.

## FUNDING INFORMATION

This work was supported by ForTra gGmbH für Forschungstransfer der Else Kröner‐Fresenius‐Stiftung (2020_EKTP12), by the José Carreras Foundation (DJCLS 03 R/2024), by research funding from the German Red Cross Blood Service Baden‐Württemberg—Hessen and particularly by the START‐Program of the Faculty of Medicine RWTH Aachen University (006/23).

## CONFLICT OF INTEREST STATEMENT

Wolfgang Wagner is cofounder of Cygenia GmbH that can provide service for various epigenetic signatures (www.cygenia.com). Apart from this, the authors have no relevant competing interests.

## ETHICS STATEMENT

All blood samples were taken after informed and written consent, in accordance with the Declaration of Helsinki, as approved by either the Ethic Committee of the Use of Human Subjects at the University of Aachen (permit number: EK 206/09; untreated donors) or by the Ethic Committee at the Medical Faculty Mannheim, Heidelberg University (AZ: 2025‐602; allogeneic donors).

## Supporting information


Appendix S1.

Appendix S2.

## Data Availability

Methylation markers were identified based on publicly available Illumina Bead Chip Microarray data. The list of GEO identifiers is provided in Tables S1 and S2. Raw data from the targeted methylation analysis by digital PCR are available upon request.
