# Supplementary material for: Monitoring of haematopoietic stem cell mobilization by targeted DNA methylation analysis
Source: Br J Haematol. 2026 Mar 17;208(6):2284–7. doi: 10.1111/bjh.70446 (PMC13267480; doi:10.1111/bjh.70446)
Supplement: Supplementary file 1 — Appendix S1. [file BJH-208-2284-s001.pdf]

Supplementary file

Monitoring of hematopoietic stem cell mobilization by targeted DNA methylation analysis

Wouter Hubens, Anke Diehlmann, Patrick Wuchter and Wolfgang Wagner

Supplemental methods

Selection of HSC specific methylation sites

We used two similar approaches to select candidate biomarkers for HSCs. Both methods relied on a low variance in DNA methylation across samples and a high difference in methylation between HSCs and other cell types. In one approach (1), we compared a balanced reference dataset from Gene Expression Omnibus (GEO) that had approximately the same sample sizes for each cell type (Supplemental Table S1). The data was processed and normalized in R (v4.1.3), using the minfi package, including single-sample Noob (ssNoob) (2). The ssNoob normalized methylation values were then ranked based on the mean β-value difference between HSPCs and the average of all other cell types, with β-diff >0.5 as hypermethylated and β-diff <0.5 as hypomethylated. From this ranking, three CpGs were arbitrarily selected that had a low variation in β-values across all samples: *SP140, MYO1D* and *STK17A*. In the second approach (3), we performed a systematic search on GEO and attempted to include all datasets of sorted blood cells that had raw IDAT files published (Supplemental Table S2). In line with the first approach, these IDAT files were loaded into R and normalized with Minfi. Next, the normalized β-values were analyzed using our recently developed CimpleG pipeline, a computation framework specifically designed for the delineation of methylation signatures (4). This pipeline evaluates how each CpG performs in classifying a specific cell type based on the area under the precision-recall curve (AUPR) from a 10-fold stratified cross-validation. The average AUPR is then weighted by multiplying the AUPR by the proportion of folds where the CpG is selected as a top scoring candidate. The top three candidate CpGs with the highest weighted AUPR were *CD48*, *NFATC1*, and *SP140*. As *SP140* was overlapping between the two approaches, this led to five candidate CpGs for further validation.

Sample processing

Leukapheresis material has a high cell density. Therefore, we diluted the blood 1:10 for DNA isolation (20µl blood with 180µl PBS) and then followed the protocol of the QIAamp DNA Mini Kit. In short, diluted samples were lysed at 56°C for 10 minutes and purified over spin columns. For a pure and high DNA yield, we performed an additional dry-spin for 3 minutes at 11.000 x g with a new collection tube and incubated with the elution buffer for 5 minutes instead of 1 minute. The DNA concentration was measured on a Nanodrop2000 (Thermofischer) and 500ng was bisulfite converted using the EZ DNA

methylation kit (Zymo Research), following the manufacturers protocol. To avoid carryover of ethanol, prior to elution, an additional dry-spin for 3 minutes at 11.000 x g was performed and the samples were incubated with 20µl elution buffer for 5 minutes.

Digital PCR

Digital PCR was performed on a QIAcuity One, 5-Plex device (Qiagen) using nanoplates with 8.5k partitions. For each sample, each CpG site was evaluated in a separate well using 2µl of bisulfite converted DNA (~50ng). Samples were heat activated at 95°C for 2 minutes followed by a 2-step cycling at 95°C for 15 seconds followed by 54°C for 30 seconds (40 cycles). Imaging was performed for the green and yellow channels at 500ms exposure and 6 gain. Analysis was performed in the QIAcuity Software Suite (v3.0.0). Threshold for positive partitions were set manually for each target based on the background fluorescence of a non-template control. The software calculated the positive copy numbers for the methylated and unmethylated DNA strands based on the positive and negative partitions using a Poisson distribution. The methylation percentage was calculated by dividing the copy number of methylated strands by the sum of methylated and unmethylated strands.

Statistical analysis

The DNA methylation values of the individual CpGs were compared to each other using Pearson correlation r with two-sided t-tests for significance. For determining if there were significant differences in methylation at these sites between G-CSF treated and untreated donors we first tested if the data was normally distributed with the Shapiro Wilk test. As the data did not pass normality ($p < 0.05$), we used a non-parametric Kruskal-Wallis test, with Benjamini and Hochberg FDR correction for multiple testing. To generate a multiple linear regression model based on the methylation values of 50 samples, we used Microsoft Excel 2016 with the Analysis toolpack add-on. These training samples were not considered for the test set to predict epigenetic cell counts against conventional cell counts. In that comparison correlation is presented as Pearson r, with the associated two-sided t tests for significance. As the model does not force positive values, any sample with a negative estimation was considered as having 0% HSCs. The predicted epigenetic counts were compared between donors with and without G-CSF treatment using the Mann-Whitney-U-Test (Shapiro Wilk test for normality of $p < 0.001$). Lastly, we performed a receiver operating characteristics (ROC) analysis using GraphPad Prism 9.0 to determine at which epigenetic cell count cut-off value, we could discern G-CSF treated and untreated donors with the highest sensitivity and specificity.

Supplemental Figures

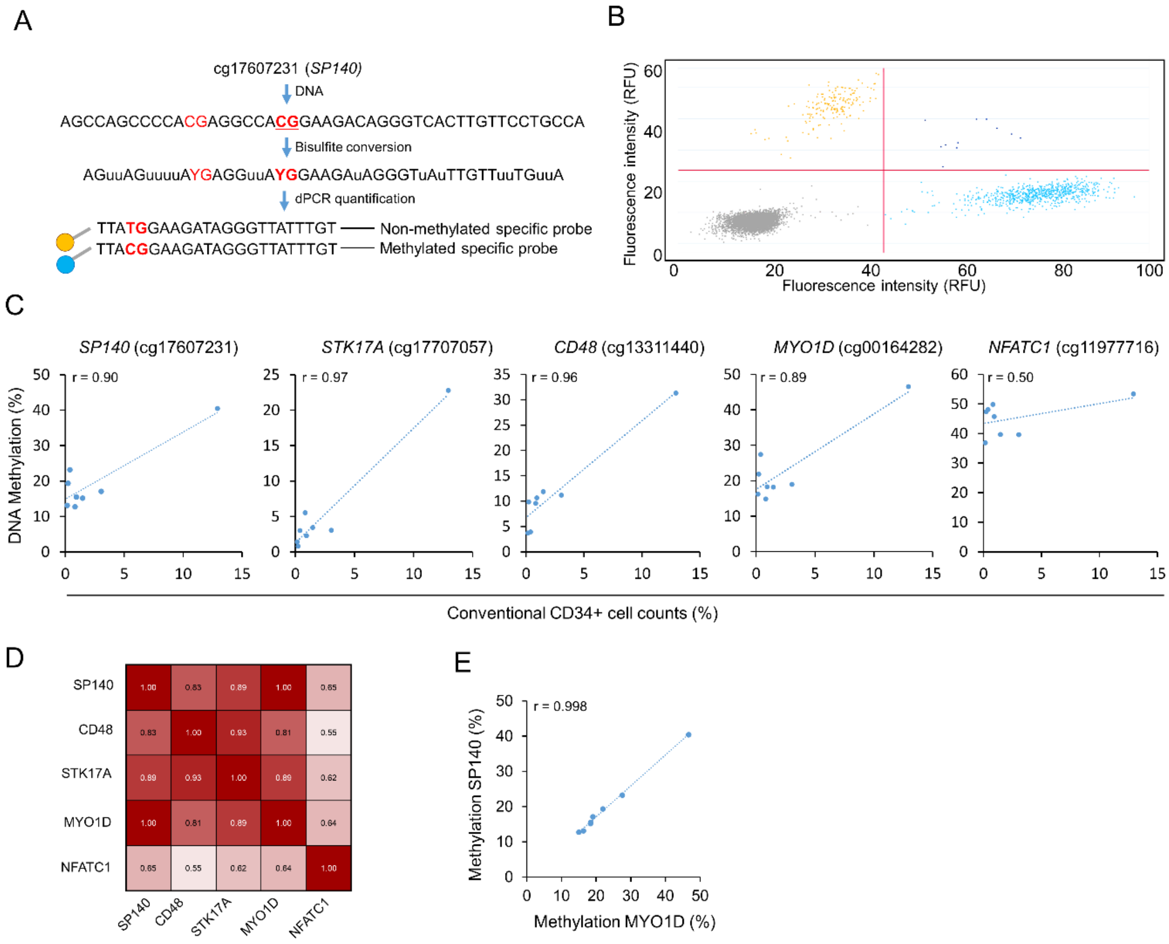

Supplemental Figure S1: Initial screening of CpG sites as epigenetic biomarkers for HSC count.

A) Schematic representation of the methodology. Isolated DNA was exposed to bisulfite to convert all unmethylated cytosines to uracil (u). During PCR amplification this uracil is replaced by thymine (T). Two fluorescently labelled probes are designed that respectively bind to either methylated CG or unmethylated TG. **B)** Representative 2D scatterplot of dPCR results for *SP140*. Partitions are quantified as negative for SP140 DNA (grey), positive for non-methylated *SP140* (yellow) or methylated *SP140* (light blue). A few partitions contain multiple *SP140* DNA strands with a mix of methylated and unmethylated (dark blue). The software uses Poisson statistics to defer copy numbers / µl based on this droplet distribution. **C)** DNA methylation levels of the 5 candidate CpGs in 8 donors with known CD34+ cell counts. Pearson correlation r is provided. **D)** Correlation matrix (Pearson r) shows that the methylation values at the five CpGs strongly correlate with each other. (**E**) Methylation of *MYO1D* is excluded from further analysis as the absolute methylation values were almost identical (Pearson r = 0.998) to *SP140*.

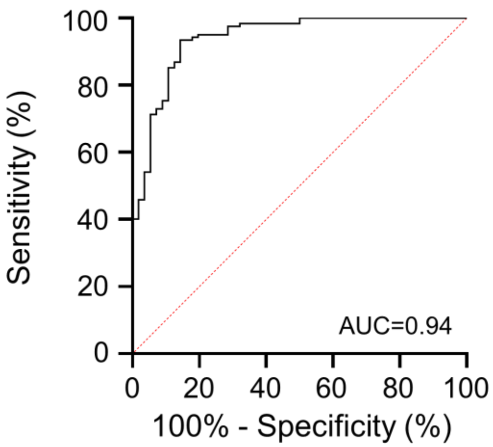

Supplemental Figure S2: Sensitivity of the epigenetic signature to identify successful mobilization.

A receiver operating characteristic analysis of epigenetic predicted cell counts classified 93% of samples correctly as having stem cell mobilization (n=113). Only 14% of untreated donors (n=8) were falsely categorized as having underwent G-CSF mobilization.

Supplemental references

1. Bocova L, Hubens W, Engel C, Koschmieder S, Jost E, Wagner W. Quantification of hematopoietic stem and progenitor cells by targeted DNA methylation analysis. Clin Epigenetics. 2023;15(1):105.
2. Fortin JP, Triche TJ, Jr., Hansen KD. Preprocessing, normalization and integration of the Illumina HumanMethylationEPIC array with minfi. Bioinformatics. 2017;33(4):558-60.
3. Hubens WHG, Maie T, Schnitker M, Bocova L, Puri D, Wessiepe M, et al. Targeted DNA Methylation Analysis Facilitates Leukocyte Counts in Dried Blood Samples. Clin Chem. 2023;69(11):1283-94.
4. Maie T, Schmidt M, Erz M, Wagner W, Costa I. CimpleG: finding simple CpG methylation signatures. Genome Biol. 2023;24(1):161.
